# The Consecutive 200 Cases of Endoscopic-Combined Intrarenal Surgery: Comparison between Standard and Miniature Surgeries

**DOI:** 10.3390/medicina59111971

**Published:** 2023-11-08

**Authors:** Young Joon Moon, Kang Su Cho, Dae Chul Jung, Doo Yong Chung, Joo Yong Lee

**Affiliations:** 1 Department of Urology, Kyungpook National University Hospital, Kyungpook National University School of Medicine, Daegu 41944, Republic of Korea; jjunny74@naver.com; 2 Department of Urology, Severance Hospital, Urological Science Institute, Yonsei University College of Medicine, Seoul 03722, Republic of Korea; 3 Department of Urology, Prostate Cancer Center, Gangnam Severance Hospital, Urological Science Institute, Yonsei University College of Medicine, Seoul 06273, Republic of Korea; kscho99@yuhs.ac; 4 Department of Radiology, Severance Hospital, Research Institute of Radiological Science, Yonsei University College of Medicine, Seoul 03722, Republic of Korea; daechul@yuhs.ac; 5 Department of Urology, Inha University College of Medicine, Incheon 22212, Republic of Korea; dychung@inha.ac.kr; 6 Center of Evidence Based Medicine, Institute of Convergence Science, Yonsei University, Seoul 03722, Republic of Korea

**Keywords:** kidney, nephrolithiasis, lithotripsy, urinary calculi

## Abstract

*Background and Objectives*: Percutaneous nephrolithotomy (PCNL) is still the gold-standard treatment for large and/or complex renal stones. Endoscopic combined intrarenal surgery (ECIRS) was developed with the goal of minimizing the number of access tracts of PCNL while simultaneously improving the one-step stone-free rate (SFR). The aim of this study was to share the experience of the consecutive 200 cases of ECIRS in one institute and analyze surgical outcomes of mini-ECIRS and standard ECIRS. *Materials and Methods*: We performed ECIRS for 200 adult patients between July 2017 and January 2020. An ECIRS was performed with the patient under general anesthesia in the intermediate-supine position. Surgeries were finished using a tubeless technique with a simple ureteral stent insertion. *Results*: There were significant differences in the mean maximal stone length (MSL), the variation coefficient of stone density (VCSD), the linear calculus density (LCD), the Seoul National University Renal Stone Complexity (S-ReSC), and the modified S-ReSC scores in stone characteristics, and estimated blood loss (EBL) and operation time in peri-operative outcomes between conventional and mini-ECIRS. After propensity-score matching, there was only a difference in EBL between the two groups. In logistic regression models, MSL [odds ratio (OR) 0.953; 95% confidence interval (CI) 0.926–0.979; *p* < 0.001], LCD (OR 4.702; 95% CI 1.613–18.655; *p* = 0.013) were significant factors for the success rate after ECIRS. *Conclusions*: In patients who underwent a mini-ECIRS, the stones were relatively smaller and less complex, and the operation time was shorter. However, if the size of stones was similar, there was no difference in the success rate, but EBL was lower in mini-ECIRS than in standard surgery.

## 1. Introduction

A renal stone is a common disease in urology and, to date, the prevalence is increasing worldwide [1]. Especially, a complex renal stone is defined as having multiple stones or anatomical or functional abnormalities. If a complex renal stone is not treated, it can cause serious complications such as renal failure, sepsis, and septic shock; thus, appropriate treatment is essential. A complex renal stone is a challenging case to manage in the urology field. In guidelines, percutaneous nephrolithotomy (PCNL) is recommended as the primary option in the treatment of large and/or complex renal stones [2,3]. However, El-Nahas AR et al. reported that the stone-free rate (SFR) of PCNL monotherapy in the treatment of staghorn stones was only 56% [4]. In the treatment of patients with staghorn stones, performing a PCNL using multiple tracts or sessions can achieve a high SFR, but there is a problem of increased related complications [5]. For this reason, after PCNL was first introduced by Fernstrom et al. in 1976 [6], various developments, such as minimally invasive PCNL, have been made to reduce complications and increase the SFR.

Endoscopic combined intrarenal surgery (ECIRS) was started with the goal of reducing complications and improving safety by reducing the number of access tracts while simultaneously increasing the SFR [7]. ECIRS was first reported by Scoffone et al. in 2008 [8,9]. It simultaneously combines PCNL and retrograde intrarenal surgery (RIRS) to explore the whole urinary tract including the renal cavities and aims to resolve urolithiasis with a one-step surgical treatment [10]. The major advantages of using a simultaneous antegrade-retrograde approach for complex renal calculi are better SFRs and lower bleeding complications by avoiding multiple tract PCNL. Until recently, many studies have been conducted and reported on clinical results and the safety of ECIRS [11]. In addition, not only is radiation exposure less than standard in PCNL, but it can also be a new alternative for treating stones in patients with renal congenital anomalies [12]. In 2014, mini-ECIRS was first reported by Hamamoto et al. [13]. Although the implementation of mini-ECIRS has increased since then, there is still a lack of studies on the indications, clinical outcomes, and complications of mini-ECIRS and comparative studies with standard ECIRS. The purpose of this study was to report the experience of the consecutive 200 cases of ECIRS in one institute and analyze the clinical outcomes of mini-ECIRS and standard ECIRS.

## 2. Materials and Methods

### 2.1. ECIRS Patient Population and Technique

We retrospectively analyzed 200 consecutive ECIRS cases between July 2017 to January 2020 at one institution (Severance Hospital, Seoul, Republic of Korea). A renal calyceal puncture was performed by an interventional radiologist using ultrasonic guidance before surgery or performed by a urologic surgeon using ultrasonic and fluoroscopic guidance during the surgery. ECIRS was performed in the intermediate-supine [Galdakao modified supine Valdivia (GMSV)] position under general anesthesia. First, an Amplatz Super Stiff guidewire was indwelled under cystoscopic guidance, and then an 11/13-Fr ureteral access sheath (Uropass; Olympus Corp., Tokyo, Japan or Navigator, Boston Scientific, Boston, MA, USA) was inserted through the guidewire. Next, the collecting system was identified using a flexible ureteroscope [LithoVue™ single-use digital flexible ureteroscope (Boston Scientific, Boston, MA, USA)] and the puncture point was visualized. If a renal calyceal puncture was not performed before surgery, an ultrasonic and fluoroscopic guidance puncture was performed by a urologist at this point. Afterwards, the formed tract was dilated with a balloon dilator or a one-step dilator. Fifty-five patients underwent a standard ECIRS using a 20-Fr nephroscope and 145 underwent a mini-ECIRS with a 12-Fr mini-nephroscope. Lithotripsy was performed with a holmium:YAG laser lithotripter (VersaPulse™ PowerSuite™ 100 W; Lumenis, Tel Aviv, Israel). All cases were performed simultaneously using a flexible ureteroscope and a tubeless technique with a simple ureteral stent indwelling.

### 2.2. Baseline Patient and Stone Characteristics

Patient characteristics, detailed history of urolithiasis, and stone characteristics including the maximal stone length (MSL), mean stone density (MSD), stone heterogeneity index (SHI), the variation coefficient of stone density (VCSD), and linear calculus density (LCD) were collected for analysis. Non-contrast computed tomography (NCCT) images of all patients were acquired and analyzed to collect stone characteristic data. MSD was defined as the mean Hounsfield units (HU) over the region of interest and was measured using magnified axial NCCT images at the level of the largest stone diameter. SHI was defined as the standard deviation of the HU for the same region of interest. Stone complexity was assessed and measured using the Seoul National University Renal Stone Complexity (S-ReSC) and modified S-ReSC scores [14,15]. The stone analysis method consisted of a quantitative analysis of the stone composition through Fourier transform infrared (FTIR) spectroscopy, and the stones were sent to the GC laboratory in Yongin, Korea, for analysis. The stones were categorized into ten groups: (1) a pure calcium oxalate (CaOx) monohydrate group (stones containing 100% of CaOx monohydrate); (2) a CaOx monohydrate ≥ 80% group; (3) a CaOx monohydrate < 80% group; (4) a mixed CaOx group (stones containing CaOx monohydrate and CaOx dehydrate); (5) a pure carbonate apatite group (stones containing 100% of carbonate apatite); (6) a struvite ≥ 80% group; (7) a struvite < 80% group; (8) a pure uric acid group (stones containing 100% of uric acid); (9) a pure cystine group (stones containing 100% of cystine); and (10) a pure brushite group (stones containing 100% of brushite). To evaluate the treatment results, complication rate and post-treatment complication data were collected including changes in hemoglobin, creatinine, and an estimated glomerular filtration rate. Successful treatment was assessed 3 months after treatment and was defined as the absence of residual stones and the presence of residual fragments sized 3 mm or less on NCCT images.

### 2.3. Statistical Analyses

In comparing the basic characteristics of patients, a Student’s two-sample *t*-test and Pearson chi-square test with Yates’ continuity correction were used. In addition, a univariate and multivariate logistic regression analysis was performed to identify factors associated with successful treatment. A Welch’s two-sample *t*-test was performed to confirm homoscedasticity between the two groups, and a Shapiro-Wilk test was performed to analyze the normality of each group. Additionally, a Mann-Whitney U test and Wilcoxon rank-sum test were performed to confirm the independence of both groups. After comparing the variances of two samples from normal populations, the presence or absence of variation equality was confirmed in a *t*-test.

To further specify the characteristics of the subjects, we performed propensity score matching. Each mini-ECIRS patient was matched 1:1 with a standard-ECIRS patient. Mini-ECIRS patients and standard-ECIRS patients each conducted a 1:1 matching, and logistic regression with multivariate analysis using MSL, MSD, SHI, VCSD, and LCD was used to calculate propensity scores. In order to present a better comparison group, propensity score matching was used to improve matching according to the stone characteristics [16]. Regarding the propensity scores between the two groups, pretreatment characteristic distribution did not differ [17].

All statistical analyses in this study were performed using R software (version 4.1.3, R Foundation for Statistical Computing, Vienna, Austria).

### 2.4. Ethics Statement 

The Institutional Review Board of our medical institution reviewed and approved the protocol for this study (Approval No. 2022-3041-002).

## 3. Results

### 3.1. Baseline Characteristics and Treatment Outcomes

Of the 200 ECIRS patients, 120 patients were male and 80 were female. The mean age was 58.49 ± 13.46 years. The MSL was 29.76 ± 14.35 mm. The average MSD was 995.12 ± 366.80 HU, and the mean SHI was 209.28 ± 104.45 HU. The VCSD was 21.61 ± 20.98 and the LCD was 0.98 ± 1.37. The S-ReSC score was 4.49 ± 2.63 and the average modified S-ReSC score was 6.68 ± 3.34. The mean operative time was 79.69 ± 32.50 min, and the patient was discharged on postoperative day 2 (IQR 1.75–3). Twenty-two patients underwent simultaneous ipsilateral ECIRS and contralateral RIRS, and 11 patients underwent ipsilateral ureteroscopic ureterolithotomy. There were significant differences in MSL, VCSD, LCD, S-ReSC, and modified S-ReSC scores in the stone characteristics, and the estimated blood loss (EBL) and operation time in peri-operative outcomes between a conventional and a mini-ECIRS (Table 1). The results of stone composition analysis were as follows: the pure CaOx monohydrate group 20%, the CaOx monohydrate ≥ 80% group 2%, the CaOx monohydrate < 80% group 2%, the mixed CaOx group 8%, the pure carbonate apatite group 6.5%, struvite ≥ 80% group 15%, the struvite < 80% group 25.5%, the pure uric acid group 19.5%, the pure cystine group 0.5%, and the pure brushite group 1%.

### 3.2. Comparison of Propensity Score-Matched Standard ECIRS and Mini-ECIRS

As a result of propensity score matching, the number of patients in each group was 37, and there were no significant differences in age (*p* = 0.499), sex (*p* = 0.811), MSL (*p* = 0.856), MSD (*p* = 0.880), or SHI (*p* = 0.927) between the two groups. Also, there were no significant differences in S-ReSC and modified S-ReSC scores between the two groups (*p* = 0.737; *p* = 0.744). After propensity score matching, only EBL showed a significant difference between the two groups, being significantly higher in a standard ECIRS than a mini ECIRS (*p* = 0.010) (Table 2).

### 3.3. Predictive Factors of Successful ECIRS 

In logistic regression models, the MSL [odds ratio (OR) 0.953; 95% confidence interval (CI) 0.926–0.979; *p* < 0.001] and LCD (OR 4.702; 95% CI 1.613–18.655; *p* = 0.013) were significant factors for success rate after ECIRS (Table 3).

## 4. Discussion

A Renal stone is a common disorder, and its prevalence has been increasing worldwide [1]. In the United States, it is reported that 5% of the population is affected by nephrolithiasis [18]. Renal staghorn stones account for 10–20% of all renal stones and are a serious disease that, if left untreated, can lead to renal failure, severe infection, septic shock, and even death. Therefore, treatment of these complex renal stones is essential to prevent complications, and as it is a challenging treatment in the field of urology, many studies are being conducted [19]. The European Association of Urology (EAU) and American Urological Association (AUA) guidelines for the management of renal calculi > 20 mm recommend PCNL as the first-line therapy [2,3,20].

PCNL was first reported by Fernstrom et al. in 1976 [6,21], and it showed relatively high SFRs for partial and complete staghorn stones [22]. As PCNL has been widely performed, various patient positions have been suggested and Ibarluzea et al. first reported PCNL using an intermediate-supine (GMSV) position in 1992 [23]. Additionally, much effort has been made to increase the stone-free rate in the treatment of complex renal stones and, as part of that effort, a multitract PCNL using multiple PCN tracts has been performed. A multitract PCNL has the advantage of relatively efficiently increasing the stone-free rate by accessing stones through various routes, but its major disadvantages are increased blood loss and renal parenchymal injury caused by the increased number of punctures. Chen et al. [19] reported a single-center experience comparing a multitract mini-PCNL and ECIRS in the treatment of renal staghorn stones. From January 2018 to September 2021, a retrospective analysis was conducted on a total of 34 patients, including 17 ECIRS patients and 17 multitract mini-PCNL patients. There was no significant difference in the stone-free rate and mean operative time between the two groups (*p* = 0.94 and *p* = 0.63, respectively). There were no significant differences between the two groups for complications including hemoglobin loss and postoperative blood transfusion rate. However, postoperative pain was significantly lower in the ECIRS group (*p* < 0.001). In conclusion, they described that both an ECIRS and multitract mini-PCNL were effective and safe in the treatment of renal staghorn stones and that an ECIRS showed less postoperative pain.

However, PCNL is not the only option for the treatment of patients with a greater stone burden. In 1992, Ibarluzea et al. [23] proposed a method of performing PCNL for the treatment of large renal stones while simultaneously removing stone fragments through an Amplatz catheter using a ureteroscope with clear vision. Improvements in surgical techniques and the development of endourologic devices have led to significant advances in the treatment of complex renal stones. RIRS plays a major role in reducing complications and improving clinical outcomes through combination with PCNL [24]. Later in 2008, Scoffone et al. [9] coined the term ECIRS and operated under the GMSV position. The SFR of ECIRS was higher than PCNL monotherapy, and due to these advantages, it has been in the spotlight as a new treatment method for complex renal stones. 

Recently, a mini-ECIRS using a mini-PCNL for a safer and more effective ECIRS has been reported. Kwon et al. [25] conducted a prospective study to analyze the feasibility of carrying out an ECIRS with a supine mini-PCNL and RIRS for patients with bilateral stones in a single session. The study was conducted on a total of 26 patients. The SFR was 76.9% and 92.3% on the ECIRS side and the contralateral RIRS side, respectively. Complications occurred in two patients, but they completely improved with appropriate medical treatment after surgery. Accumulation of surgical experience (OR 117.3, *p* = 0.046) was a significant predictor of stone-free status. They concluded that carrying out an ECIRS and contralateral RIRS in a single session for patients with bilateral stones is feasible and safe.

In our prior study, we retrospectively analyzed the first 100 cases of ECIRS in Korea and compared the outcomes with shock-wave lithotripsy (SWL) [26]. ECIRS had a higher SFR and success rate compared to SWL. As in this study, a multivariate logistic regression analysis was performed to identify predictors of successful ECIRS, and the results showed that smaller stone size (OR: 0.947, *p* = 0.002) and lower S-ReSC score (OR: 0.759, *p* = 0.011) were independent predictors of successful ECIRS. 

Until recently, many studies have compared clinical outcomes of ECIRS and PCNL for complex renal stones. Wen et al. [27] conducted a systematic review and meta-analysis to analyze the clinical outcomes of a simultaneous mini-PCNL combined with flexible ureteroscopy as a single-stage therapy to treat the partial staghorn calculi and compared it with conventional mini-PCNL monotherapy. Sixty-seven patients with partial renal staghorn stones were randomly divided into the mini-PCNL group and the ECIRS group, respectively. The ECIRS group showed a significantly higher one-step SFR (87.88% vs. 58.82%, *p* = 0.007) than the mini-PCNL group, and there was no statistical difference in clinical complications between the two groups (*p* = 0.409). They concluded that the simultaneous combined mini-PCNL and flexible ureteroscopy was more effective and safer than conventional mini-PCNL monotherapy in the treatment of partial staghorn stones. Gauhar et al. [28] conducted a systematic review and meta-analysis comparing ECIRS and conventional PCNL in the treatment of renal stones. Analysis was conducted on a total of 2054 patients from 17 studies, including 800 ECIRS patients and 1254 conventional PCNL patients. A meta-analysis was performed on four categories of parameters: surgical time and length of stay, bleeding, infection complications, stone-free rate, and retreatment. There was no significant difference in surgical time, mean postoperative length, blood transfusion rate, and postoperative sepsis between the two groups (*p* = 0.20, *p* = 0.69, *p* = 0.15, and *p* = 0.25, respectively). The mean hemoglobin drop and retreatment rate were lower in the ECIRS group (*p* = 0.03, *p* = 0.002, respectively), and the stone-free rate was significantly higher in the PCNL group (*p* < 0.0001). In conclusion, although conventional PCNL showed a higher stone-free rate, ECIRS showed a shorter operative time, lower complication rate, and retreatment, thus, supporting the contention that ECIRS is an effective and safe treatment method.

There was a study comparing the treatment outcomes between a standard ECIRS and a mini-ECIRS. Usui et al. [29] retrospectively analyzed 77 patients who underwent a single-session mini ECIRS and 77 patients who underwent a single-session standard ECIRS between April 2009 and May 2016. The SFR was similar for a mini-ECIRS and a standard ECIRS at 61.1% and 52.0% (*p* = 0.388), respectively. In terms of postoperative complications, Clavien-Dindo classification grade 2 or higher complications were 19.5% and 26.0% in the mini-ECIRS and standard ECIRS groups, respectively (*p* = 0.442), and severe complications of Clavien-Dindo classification grade 3 or higher were 2.6% and 3.9%, respectively (*p* > 0.99). There was no significant difference between the two groups. In addition, there was no significant difference in bleeding-related complications at 2.6% and 6.5% (*p* = 0.442), respectively, but pseudoaneurysm and significant blood loss were not observed in the mini-ECIRS group. They concluded that compared with a standard ECIRS, a mini-ECIRS has the potential to reduce surgery-related pain and bleeding-related complications without increasing perioperative complications while maintaining the SFR.

In our study, ECIRS was performed under the intermediate-supine (GMSV) position. Recently, many studies have been conducted and reported on patient position when performing ECIRS. Wang et al. [30] reported their experience with ECIRS using a modified prone split-leg position. Analysis was conducted on a total of 96 patients from September 2017 to January 2021. The stone-free rate was 78.1%, and the complication rate was 7.1%. Most complications were Clavien-Dindo classification grade 1 or 2, and no Clavien-Dindo classification grade 3 complications were observed. They also analyzed the risk factors associated with the ECIRS stone-free rate and found that the number of calyces in which the stone was located was the only significant risk factor (*p* = 0.01). In conclusion, they stated that ECIRS using a modified prone split-leg position is effective and safe for the treatment of renal stones. In addition, the ease of the retrograde approach was cited as an advantage of ECIRS through modified prone split-leg position.

In order to reduce complications when performing ECIRS, it is important to reduce intraoperative EBL. To reduce EBL during surgery, the renal calyceal puncture process is important. In our study, a renal calyceal puncture was performed under ultrasonic and fluoroscopic guidance. Inoue et al. [31] reported the wideband Doppler ultrasound-guided puncture for safe renal calyceal puncture. They performed a retrospective analysis on a total of 41 patients who underwent a mini-ECIRS using wideband Doppler ultrasound for the treatment of renal stones larger than 30 mm. A renal calyceal puncture using wideband Doppler ultrasound was successfully performed in all cases. The initial stone-free rate was 73.2% and the final stone-free rate after additional treatment was 97.5%. The mean hemoglobin drop was 0.54 ± 0.65 g/dL and three patients developed fever after surgery. However, no major complications such as injury to adjacent organs occurred. Univariate analysis was performed to identify risk factors for severe bleeding (hemoglobin drop of ≥1 g/dL) and, as a result of the analysis, there were no significant risk factors. They concluded that a mini-ECIRS using wideband Doppler ultrasound-guided renal puncture could be a safe and effective treatment option for the treatment of large renal stones. Next, nephrostomy tract dilation is also an important process for reducing EBL during surgery. In our study, nephrostomy tract dilation was performed in one step using a balloon dilator or a one-step dilator. The nephrostomy tract dilation procedure can be performed using either a multi-step serial dilation technique or a single-step dilation technique. However, it has not yet been proven which of the two techniques is superior in terms of safety. Gupta et al. [32] reported a prospective study comparing the single-step dilation technique and serial dilation technique when performing PCNL. A study was conducted on a total of 100 patients, including 50 patients using the single-step dilation technique and 50 patients using the serial dilation technique. The mean access tract dilation time and mean total fluoroscopy time were significantly shorter in the one-step dilation group (*p* < 0.0001). However, there was no significant difference in the requirement of blood transfusion and post-operative complications rate between the two groups. They concluded that the single-step dilation technique showed clinically comparable results when compared to the conventional serial dilation technique and was therefore a safe, effective, and economical method. They also recommended that the method be selected according to the surgeon’s preference and experience.

Recently, a method using an antegrade flexible ureteroscopy to increase the stone-free rate when performing ECIRS was reported. Yang et al. [33] introduced a new technique named the ‘Through-through’ approach. The process of this technique is to first use a nephroscope to identify the orientation of the target calyx and then insert a flexible ureteroscope through the nephroscope instrument channel to identify and remove residual stones. They performed a retrospective analysis on a total of 68 patients who underwent ECIRS using a ‘Through-through’ approach. The mean operative time was 100.1 ± 18.0 min, and the stone-free rate was 91.2%. Complications of Clavien-Dindo classification grade 3 or higher were not observed, and no patients required a blood transfusion after surgery. Ten patients developed fever after surgery, but none developed septic shock. Based on these results, they concluded that ECIRS using a ‘Through-through’ approach is a safe and effective treatment method for the treatment of complex renal stones.

Recently, with the advancement of science and technology, many changes have occurred in medical care, and new medical technologies are being introduced as robotic technology is incorporated into medicine. Robotic technology is also being introduced in endourology, and a study on the treatment of renal stones using robotic technology is also being published. Tokatli et al. [34] reported a retrospective study on the treatment of complex renal stones by robot-assisted mini-ECIRS using Avicenna Roboflex™ (Elmed, Turkey). The study was conducted on a total of 42 patients. The stone-free rate was 95.5%, and complications were observed in 7.1%, and all complications were Clavien-Dindo classification grade 1. In conclusion, they stated that robot-assisted mini-ECIRS is effective and safe in the treatment of complex renal stones. In addition, the possibility of endoscopic flexible ureteroscopic evaluation of the collecting system at the end of surgery was described as an important advantage of robot-assisted mini-ECIRS. Although additional research is necessary in the future, ECIRS using a robotic system is also considered to be a good option for the treatment of renal stones.

What is unique about our study is that this is the first study to compare surgical outcomes between a standard ECIRS and a mini-ECIRS. We analyzed the surgical outcomes in the consecutive 200 cases of ECIRS and compared a standard ECIRS and mini-ECIRS by performing propensity score matching using MSL, MSD, SHI, VCSD, and LCD. Also, we performed logistic regression analyses to identify factors significantly associated with successful treatment. This study result may be helpful for urologists in selecting the management of renal stones.

Our study had several limitations. First, complications between standard ECIRS and mini-ECIRS were not compared. Analysis of complications is an important element in evaluating the safety of each procedure, so analysis will be necessary in future studies. Second, there was no classification or analysis of the location and configuration of the renal stones, which may affect the success rate. Instead, we evaluated S-ReSC and modified S-ReSC scores that can predict the SFR after PCNL and RIRS, respectively. Since the S-ReSC score indicates the distribution complexity of renal stones, it is thought that it can overcome this limitation to some extent. In addition to S-ReSC, there are several scoring systems such as the Stone-Tract length-Obstruction-Number of involved calyces, and Essence of stone (S.T.O.N.E.) nephrolithometry, Guy’s stone score (GSS), and the Clinical Research Office of the Endourological Society (CROES) nomogram; however according to previous studies, there was no significant difference in predictive accuracy [35]. Regarding the configuration of stones, it is believed that more significant results can be obtained if the stones are classified into purely calyceal stones, purely pelvic stones, pelvic component-dominant complex stones, and calyceal component-dominant complex stones, and then compared and analyzed. Third, in this study, a comparative analysis of the treatment outcomes and stone characteristics was performed, but a comparative analysis of patient-reported outcomes was not performed. Patient’s subjective satisfaction with the treatment method is a very important factor. We believe that more reliable results can be obtained if data on patient-reported results are collected through questionnaires and further analyzed. Fourth, a comparative cost analysis of the two treatment methods has not been performed. Cost is an important factor to consider when deciding on a treatment method. Additional cost analysis will be helpful in selecting a treatment method for renal stones. Fifth, a relatively short follow-up period is also a limitation of this study. Regarding the outcome of treatment, the recurrence rate is an important evaluation factor, and a longer follow-up period is needed to analyze recurrence. Lastly, a small sample size and retrospective analysis are limitations of our study. In the future, a prospective randomized trial with a larger sample size and longer follow-up period could further increase the reliability of our study results.

## 5. Conclusions

In patients who underwent a mini-ECIRS, the stones were relatively smaller and less complex and operation time was shorter. However, if the sizes of the stones were similar, there was no difference in success rate, but EBL can be lower in a mini-ECIRS than in a standard surgery.

## Figures and Tables

**Table 1:** Baseline characteristics and treatment outcomes of standard ECIRS vs. mini-ECIRS.

	Total	Standard ECIRS	Mini-ECIRS	*p*-Value
No.	200	55	145	
Age (yr)	58.49 ± 13.46	57.75 ± 16.17	58.77 ± 12.32	0.671
Sex				1.000
Female	80 (40.00%)	22 (40.00%)	58 (40.00%)	
Male	120 (60.00%)	33 (60.00%)	87 (60.00%)	
BMI (kg/m^2^)	24.93 ± 5.26	24.77 ± 3.06	24.99 ± 5.89	0.732
MSL (mm)	29.76 ± 14.35	36.95 ± 14.93	27.04 ± 13.18	<0.001
MSD (HU)	995.12 ± 366.8	1047.05 ± 404.92	975.43 ± 350.75	0.218
SHI (HU)	204.28 ± 104.4	188.80 ± 103.91	210.15 ± 104.41	0.198
VCSD	21.61 ± 20.98	17.34 ± 6.62	23.23 ± 24.13	0.008
LCD	0.98 ± 1.37	0.57 ± 0.37	1.13 ± 1.57	<0.001
S-ReSC	4.49 ± 2.63	5.62 ± 2.33	4.06 ± 2.61	<0.001
MoS-ReSC	6.68 ± 3.34	7.82 ± 3.27	6.25 ± 3.28	0.003
Multiplicity				1.000
No	38 (19.00%)	10 (18.18%)	28 (19.31%)	
Yes	162 (81.00%)	45 (81.82%)	117 (80.69%)	
Bilateral				0.276
No	128 (64.00%)	39 (70.91%)	89 (61.38%)	
Yes	72 (36.00%)	16 (29.09%)	56 (38.62%)	
EBL	27.88 ± 71.53	56.27 ± 110.78	17.10 ± 45.21	0.014
Operation time	79.69 ± 32.50	91.67 ± 36.62	75.15 ± 29.68	0.001
Success				0.158
No	24 (12.00%)	10 (18.18%)	14 (9.66%)	
Yes	176 (88.00%)	45 (81.82%)	131 (90.34%)	

ECIRS, endoscopic combined intrarenal surgery; MSL, maximal stone length; MSD, mean stone density; HU, Hounsfield units; SHI, stone heterogeneity index; VCSD, Variation coefficient of stone density; LCD, linear calculus density; S-ReSC, Seoul National University Renal Stone Complexity; MoS-ReSC, modified S-ReSC; EBL, estimated blood loss. Data are presented as means ± standard deviations, numbers (percentage).

**Table 2:** Baseline characteristics and treatment outcomes of standard ECIRS vs. mini-ECIRS groups after propensity score matching using MSL, MSD, SHI, VCSD, and LCD.

	Total	Standard ECIRS	Mini-ECIRS	*p*-Value
No.	74	37	37	
Age (yr)	57.99 ± 13.82	59.08 ± 15.62	56.89 ± 11.86	0.499
Sex				0.811
Female	28 (37.84%)	15 (40.54%)	13 (35.14%)	
Male	46 (62.16%)	22 (59.46%)	24 (64.86%)	
BMI (kg/m^2^)	25.40 ± 3.56	24.84 ± 3.05	25.95 ± 3.97	0.182
MSL (mm)	30.48 ± 11.87	30.74 ± 10.22	30.23 ± 13.46	0.856
MSD (HU)	1010.95 ± 355.8	1017.24 ± 343.57	1004.66 ± 372.31	0.880
SHI (HU)	191.09 ± 100.39	192.18 ± 99.85	190.01 ± 102.29	0.927
VCSD	18.23 ± 6.38	18.32 ± 6.76	18.13 ± 6.06	0.895
LCD	0.70 ± 0.39	0.68 ± 0.39	0.71 ± 0.39	0.733
S-ReSC	4.69 ± 2.40	4.78 ± 2.03	4.59 ± 2.74	0.737
MoS-ReSC	6.85 ± 3.17	6.73 ± 3.03	6.97 ± 3.35	0.744
Multiplicity				0.247
No	15 (20.27%)	10 (27.03%)	5 (13.51%)	
Yes	59 (79.73%)	27 (72.97%)	32 (86.49%)	
EBL	39.32 ± 94.77	67.84 ± 125.50	10.81 ± 28.12	0.010
Operation time	79.95 ± 28.34	82.59 ± 30.32	77.30 ± 26.37	0.425
Success				1.000
No	12 (16.22%)	6 (16.22%)	6 (16.22%)	
Yes	62 (83.78%)	31 (83.78%)	31 (83.78%)	

ECIRS, endoscopic combined intrarenal surgery; MSL, maximal stone length; MSD, mean stone density; HU, Hounsfield units; SHI, stone heterogeneity index; VCSD, Variation coefficient of stone density; LCD, linear calculus density; S-ReSC, Seoul National University Renal Stone Complexity; MoS-ReSC, modified S-ReSC; EBL, estimated blood loss. Data are presented as means ± standard deviations, numbers (percentage).

**Table 3:** Logistic regression analyses of factors associated with successful ECIRS.

Variables	Odds Ratio	95% CI	*p*-Value
Age	1.016	0.984–1.048	0.318
BMI	0.982	0.919–1.070	0.602
MSL	0.953	0.926–0.979	<0.001
MSD	0.999	0.997–1.000	0.171
SHI	0.999	0.995–1.004	0.841
VCSD	1.014	0.988–1.071	0.573
LCD	4.702	1.613–18.655	0.013
S-ReSC	0.865	0.738–1.012	0.069
MoS-ReSC	0.890	0.781–1.011	0.075
Mini-ECIRS	2.080	0.843–4.983	0.103

ECIRS, endoscopic combined intrarenal surgery; MSL, maximal stone length; MSD, mean stone density; SHI, stone heterogeneity index; VCSD, Variation coefficient of stone density; LCD, linear calculus density; S-ReSC, Seoul National University Renal Stone Complexity; MoS-ReSC, modified S-ReSC.

## Data Availability

Data are available upon request to the corresponding author.
